# Risk factors for pregnancy-related clinical outcome in myasthenia gravis: a systemic review and meta-analysis

**DOI:** 10.1186/s13023-022-02205-z

**Published:** 2022-02-16

**Authors:** Manqiqige Su, Xiaoqing Liu, Liang Wang, Jie Song, Zhirui Zhou, Sushan Luo, Chongbo Zhao

**Affiliations:** 1 Huashan Rare Disease Center, Department of Neurology, Huashan Hospital, Fudan University, 200040 Shanghai, China; 2 National Center for Neurological Disorders, Shanghai, 200040 China; 3 Inner Mongolia Medical University, 010110 Inner Mongolia, China; 4 Radiation Oncology Center, Huashan Hospital, Fudan University, Shanghai, 200040 China

**Keywords:** Myasthenia gravis, Pregnancy, Worsening, Risk factor, Meta-analysis

## Abstract

**Objective:**

Myasthenia gravis (MG) is an autoimmune disorder that frequently affects females at reproductive age. Herein, we aimed to assess the associations of clinical factors with pregnancy-related outcome in MG.

**Methods:**

We searched PubMed and EMBASE for case–control and cohort studies that reported the MG status during or after pregnancy and relevant clinical variables. The data was extracted in proportions and odds ratios (ORs) with 95% confidence intervals (CIs) in subsequent meta-analysis.

**Results:**

Fifteen eligible articles reporting on 734 pregnancies with 193 worsening and 51 improved episodes were included out of 1765 records. The estimated worsening proportions in total, antepartum and postpartum periods were 0.36 (95% CI 0.25–0.40), 0.23 (95% CI 0.14–0.34) and 0.11 (95% CI 0.04–0.22) respectively. The proportion of pregnancy-related improvement in enrolled patients was 0.28 (95% CI 0.17–0.40), with 0.07 (95% CI 0.00–0.28) during pregnancy and 0.14 (95% CI 0.02–0.34) after pregnancy. No significant associations were disclosed between the clinical factors and MG worsening. Thymectomy before delivery is a strong predictor for MG improvement in postpartum period (OR 4.85, 95% CI 1.88–12.50, p = 0.001).

**Conclusion:**

The total proportion of pregnancy-related MG worsening and improvement in MG was 0.36 (95% CI 0.25–0.40) and 0.28 (95% CI 0.17–0.40), respectively. Thymectomy before the delivery may aid in clinical improvements associated with pregnancy. Future prospective cohort studies are required to determine more relevant factors.

**Supplementary Information:**

The online version contains supplementary material available at 10.1186/s13023-022-02205-z.

## Introduction

Myasthenia gravis (MG) is an autoimmune disorder in neuromuscular junction with a prevalence ranged from 15 to 179 per million person [1, 2]. MG has a bimodal age pattern of incidence, with a peak in female individuals under age 40, that overlaps with the reproductive age [3, 4]. During the pregnancy period with maternal physiological adaption and obstetric complications, female patients with autoimmune diseases may be more vulnerable to clinical and life events [5–9]. Characterized by fluctuating weakness in ocular, limb and bulbar muscle, rapid clinical worsening can be a serious life-threatening condition for both mothers and fetus. The optimal managements of MG during and after pregnancy require special attentions and individualized consideration.

The overall proportion for clinical worsening ranged from 10 to 90% in MG patients with pregnancy, with varied distributions in 1st, 2nd, 3rd trimester or puerperium [10–13]. A short disease duration and advanced clinical severity were associated with a profound probability for MG worsening [11–14]. However, most studies did not identify any significant correlations between duration, age at pregnancy, serological tests, delivery mode, or therapies and clinical worsening. Remarkably, the effects of thymectomy on the clinical outcome during or after pregnancy are still controversial [11, 15, 16].

To date, there is no comprehensive meta-analysis on risk or protective factors for the course of MG during/after pregnancy. Given the current inconclusive results, our review mainly aims to assess the clinical status in female MG patients during or after pregnancy, and explore the associations between the clinical factors with MG exacerbation or improvement. Herein, we conducted a systemic review and meta-analysis to estimate the overall proportion for MG worsening related with pregnancy and to identify risk factors that predict pregnancy-related clinical outcome.

## Method

The systematic review was reported according to the PRISMA (The Preferred Reporting Items for Systematic reviews and Meta-Analysis) statement [17]. Ethical permission was not required by the author’s institution for systematic reviews of available primary literature. The protocol was registered with PROSPERO, the international prospective register of systematic review (CRD42021269938).

### Search strategy

To identify all cases who had an episode of MG worsening and improvement during/after pregnancy, we searched PubMed and Embase with a last update on 12 August, 2021 with search terms including “myasthenia”, “myasthenia gravis”, “pregnancy”, “pregnant”, “labor”, “labour”, “maternal” and “gestation”. Reference lists of articles were also examined, and full-text papers were accessed and analyzed by all authors.

### Inclusion criteria

Studies eligible for the systemic review and meta-analysis met the pre-defined criteria: (1) case–control or cohort studies; (2) MG was diagnosed before or during pregnancy with supportive evidence from clinical, neurophysiological or serological testing; (3) Full description of MG status before/during/after pregnancy can be clearly identified; (4) a publication written in English to permit easy access to the source information of all included articles.

### Exclusion criteria

Articles were excluded based on the following exclusion criteria: (1) less than 5 cases included; (2) publication out of scope such as single case reports, case series, reviews, conference abstracts, commentary editorials or letters that reported no new data.

### Data extraction and quality assessment

Included studies were scrutinized for potential clinical data of myasthenia outcomes related to pregnancy. For the initial screening, the studies were independently evaluated by two authors (MS and XL) for inclusion. The conflicts if any were resolved after mutual discussions with the third author (SL). When studies included the overlapping data from the same patient sources, data from the largest cohort will be retained for further analysis. The following data were also extracted in a standard form from each study: name of the first author, year of publication, country, study design, number of patients, number of pregnancies, number of miscarriages, times of MG worsening and improvement, definition of worsening and improvement (Additional file 1). Other relevant clinical details on worsening and improvement were further screened to calculate the proportion/OR and 95% CI if available. If the OR is greater than 1 and p value is less than 0.05, then this clinical factor and MG worsening/improvements are considered to be significantly associated. Quality of evidence was accessed using the Newcastle Ottawa scale (Additional file 2), in which higher scores indicated higher quality.

### Primary and secondary outcomes

The primary outcomes were defined as clinical changes indicating MG worsening related to pregnancy. According to the previous studies [11, 12, 15, 18–20] and existing guidelines [21], the worsening of myasthenia gravis related to pregnancy was determined if: (1) newly involved muscle domain or persistent exacerbation in original involved muscle at the same drug dosage; (2) increased dosages of medications or requirement for adding a new drug to control symptoms; (3) higher stage according to correlated clinical classifications/ scoring systems; (4) clearly described as “exacerbation” or “worsening” in original articles. We evaluated the primary outcomes using the odds ratios (ORs) by possible risk factors, and estimated the pool estimates of worsening proportions during every trimester of pregnancy (0–3 months, 4–6 months and 7–9 months), and after delivery/abortion (0–6 months).

The secondary outcomes were defined as clinical changes indicating MG improvement related to pregnancy. MG improvement was defined as: (1) improvement of clinical myasthenic manifestations or a significant reduction in MG medications than those they received before pregnancy; (2) clearly described as “improved” or “better” in original articles (Additional file 3).

### Statistical analysis

Proportions and ORs were extracted and calculated based on raw data from primary studies. Freeman-Turkey double arcsine transformation method was used to calculate the combined proportion of worsening in different and the 95% confidence interval (CI). Q test and I^2^ statistics were used in detection for heterogeneity. The fixed effect analysis was applied if there exists heterogeneity (p > 0.10 and I^2^ < 50%), otherwise the random effects analysis was used. Statistical significance was set at p < 0.05 with a 95% CI. Statistical tests of Funnel plots were conducted to visualize the publication bias (N > 10) and Egger’s test was used to assess funnel plot asymmetry. Trim and fill methods were then used to detect symmetry. To evaluate the stability of results and explain the possible source of heterogeneity (N > 2), sensitivity analysis for potential risk factors was performed using the leave-one-out analysis by omitting one study at a time. A quantitative synthesis of the collective findings was performed through R language (v. 4.1.1) (http://www.r-project.org) with the meta package [22].

## Result

### Studies retrieved and characteristics

**Fig. 1:**
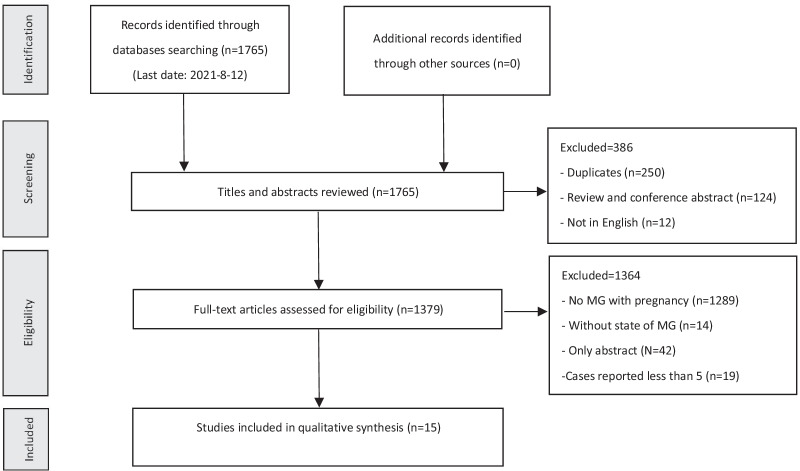
PRISMA (The Preferred Reporting Items for Systematic reviews and Meta-Analyses) flow chart of study selection

Various database searches resulted in 1765 records, of which 386 had to be excluded on the basis of titles or abstracts and 1379 were identified for full-text review. Finally, we identified 15 cohort studies that fulfilled the inclusion criteria and included in qualitive synthesis (Fig. 1) [10–16, 18, 20, 23–28], and there existed no overlapping data among these studies. The analysis enrolled 734 pregnancies in total from 574 pregnant MG patients, among who 194 pregnancies correlated with worsening and 51 pregnancies correlated with improvement.

### Primary outcome

**Fig. 2:**
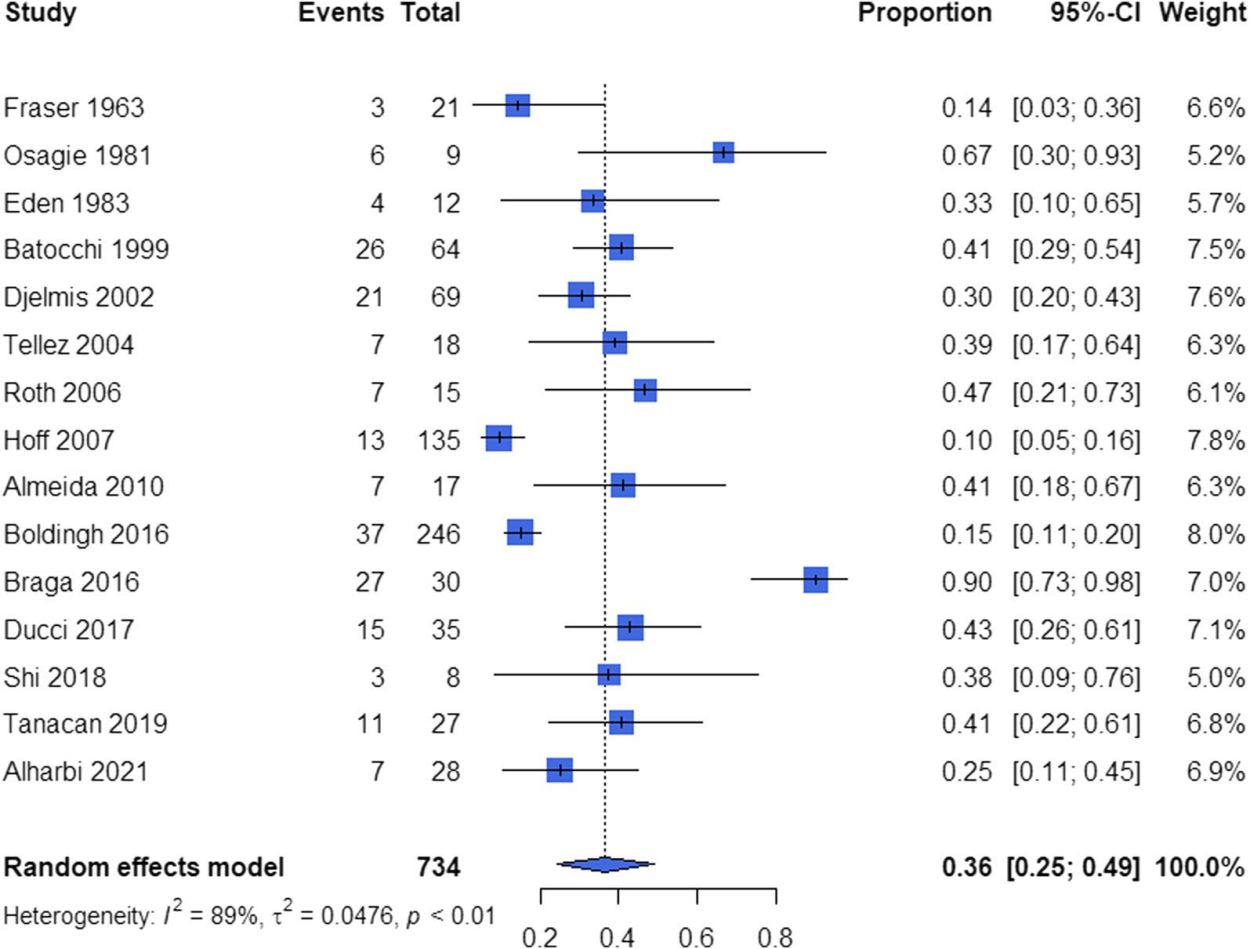
The pooled total proportions of worsening associated with pregnancy. Events: number of pregnancies with worsening associated with pregnancy. Total: total number of pregnancies

The pooled estimate of overall MG worsening proportion is 0.36 (95% CI 0.25–0.49) (Fig. 2), with 0.23 (95% CI 0.14–0.34) during pregnancy and 0.11 (95% CI 0.04–0.22) after pregnancy (N = 15). Among them, 9 studies had provided detailed proportions of worsening in each trimester from a total of 500 pregnancies and 143 episodes of MG worsening. MG worsening proportions in each trimester were then evaluated as: 0.04 in first trimester (95% CI 0.01–0.10), 0.04 in second trimester (95% CI 0.00–0.10) and 0.09 in third trimester (95% CI 0.02–0.19).

**Fig. 3:**
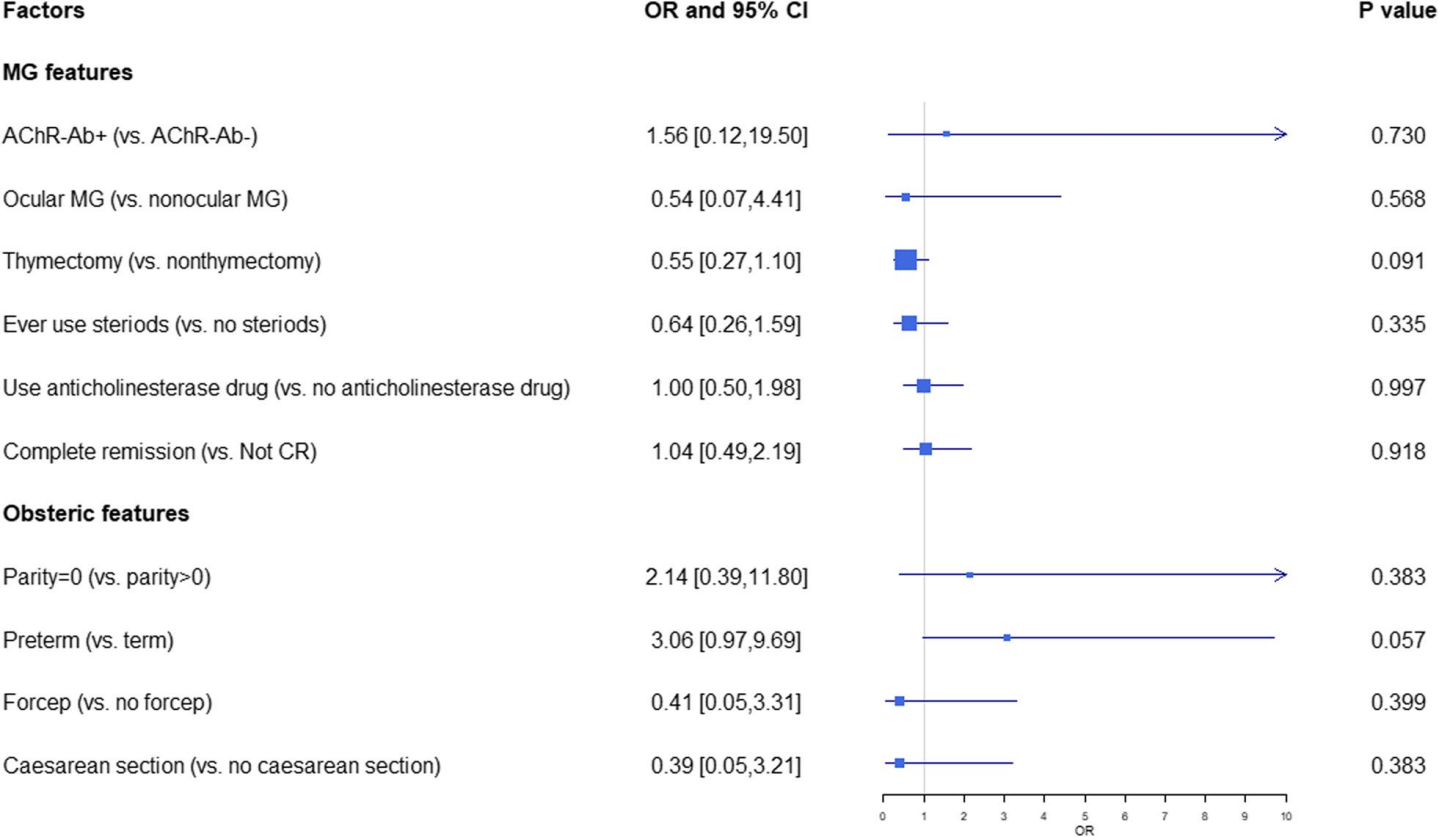
Forest plots of association between clinical factors and worsening of MG status in odds ratio. Ten risk factors were extracted and there was no significant difference in ORs for worsening

After excluding 14 factors which have very limited data for evaluation (Additional file 4), we included ten factors in the meta-analysis as potential risk factors for MG worsening related to pregnancy. These factors included thymectomy before/during pregnancy (OR 0.55, 95% CI 0.27–1.10) (N = 7), steroids use before pregnancy (OR 0.64, 95% CI 0.26–1.59) (N = 3), preterm delivery (OR 3.06 95% CI 0.97–9.69) (N = 6), parity history (OR 2.14, 95% CI 0.39–11.80) (N = 2), ocular MG (OR 0.54, 95% CI 0.07–4.41) (N = 3), delivery with forceps (OR 0.41, 95% CI 0.05–3.31) (N = 2), complete remission before pregnancy (OR 1.04, 95% CI 0.49–2.19) (N = 5), caesarean section (OR 0.39, 95% CI 0.05–3.21) (N = 4), anticholinesterase treatments before pregnancy (OR 1.00 95% CI 0.50–1.98) (N = 4), and AChR Antibody-positive (OR 1.56 95% CI 0.12–19.50) (N = 2). However, none of these factors showed significant associations with MG worsening (Fig. 3).

### Secondary outcome

**Fig. 4:**
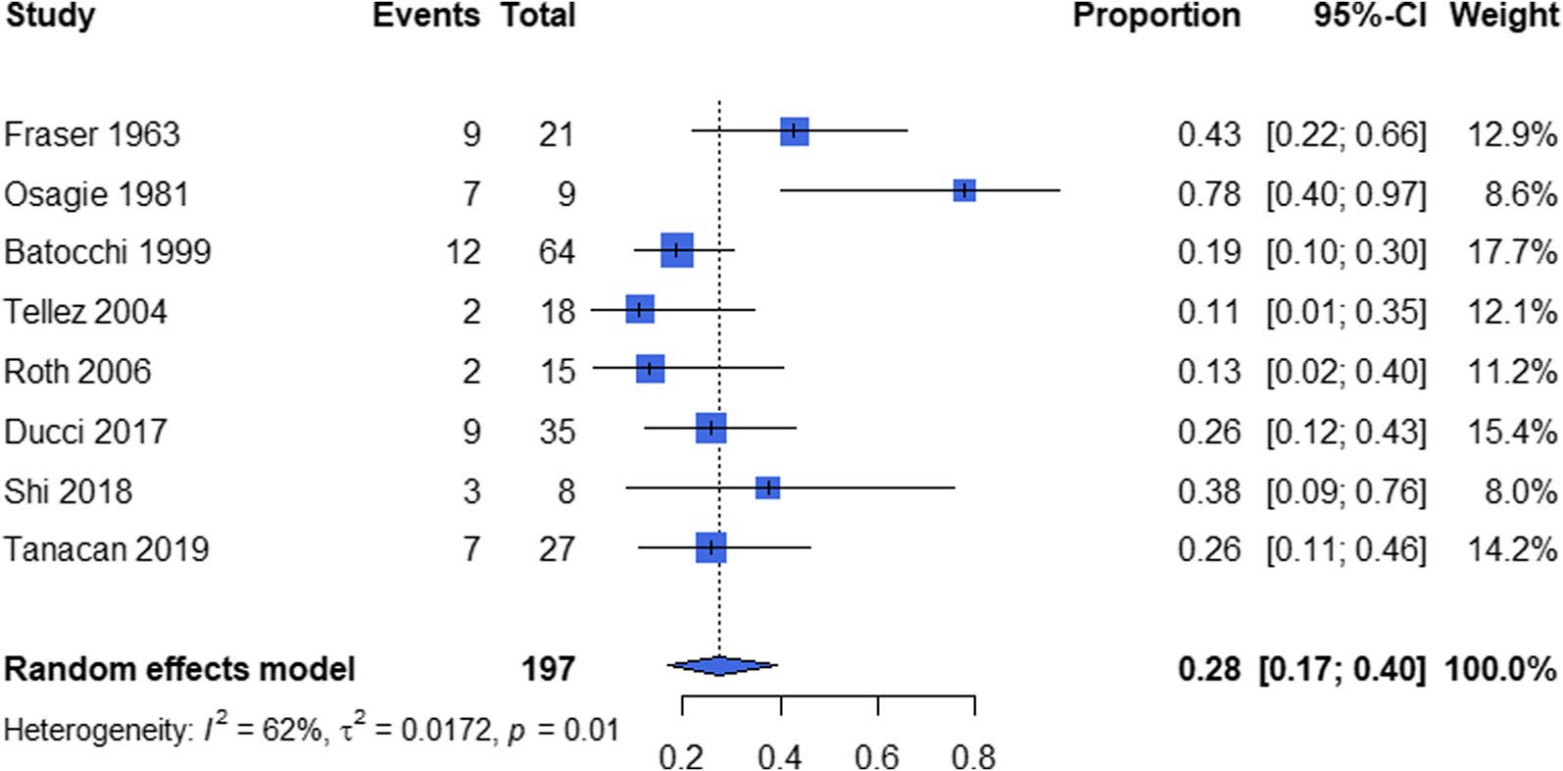
The pooled total proportions of MG improvement associated with pregnancy. Events: number of pregnancies with improved associated pregnancy. Total: total number of pregnancies

**Fig. 5:**
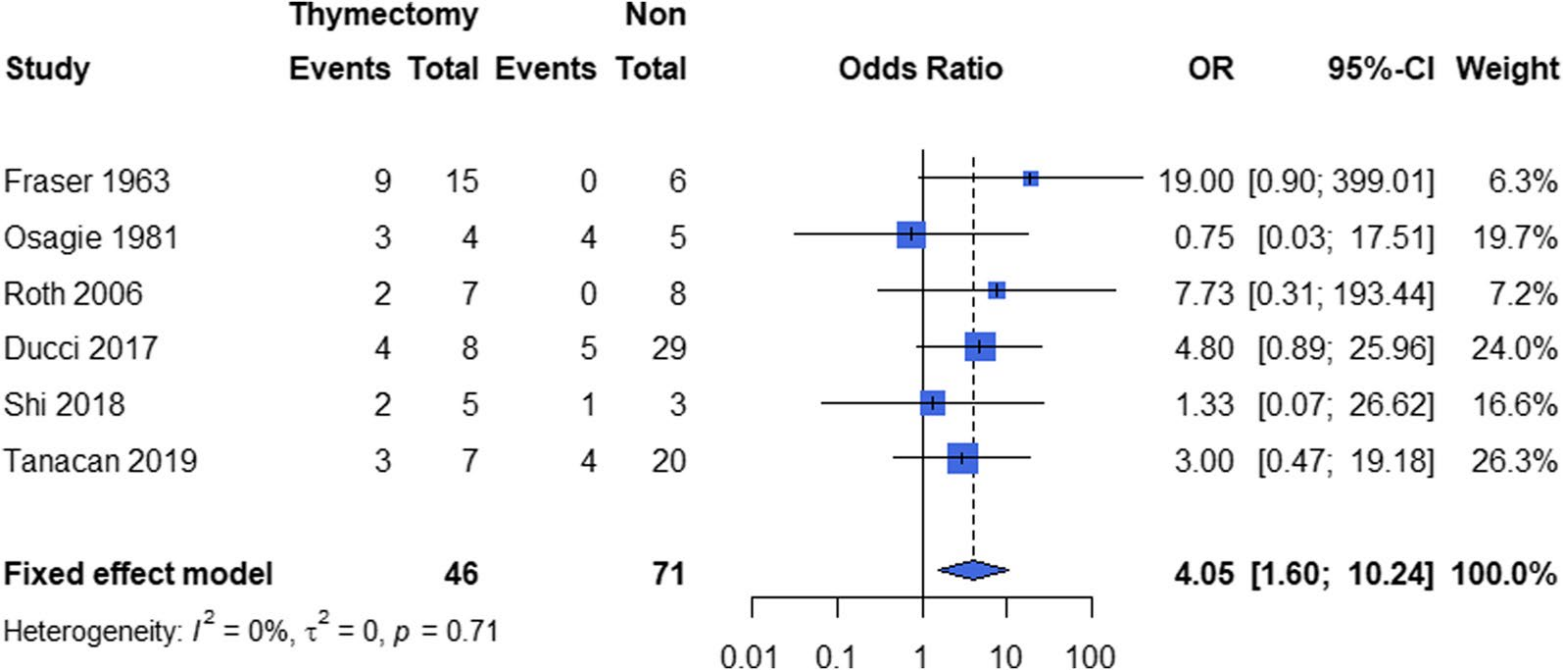
Forest plot of association between thymectomy during/after pregnancy and improvement of MG status. Events: number of pregnancies with improved associated pregnancy. Total: total number of pregnancies

The pooled proportion of pregnancy-related improved MG was 0.28 (95% CI 0.17–0.40) (N = 8) (Fig. 4), with 0.07 (95% CI 0.00–0.28) (N = 5) during pregnancy and 0.14 (95% CI 0.02–0.34) (N = 5) after pregnancy. In the meta-analysis for improved MG status during/after pregnancy, we found that the patients with thymectomy before/during pregnancy (OR 4.05, 95% CI 1.60–10.24, p = 0.003) (N = 6) has a high probability to achieve MG improvement (Fig. 5), whereas term delivery didn’t contribute in the improvement (OR 1.08, 95% CI 0.14–8.39) (N = 2) in a significant level. Other potential risk factors could not be pooled due to the limited data retrieved (Additional file 5).

### Publication bias in estimating the reported worsening episodes

As publication bias was detected by funnel plot and Egger’s test in the total proportion of pregnancy-related worsening (p = 0.009, N = 15), the trim and fill method was applied to analyze the publication bias following original funnel plots (Additional file 6). In comparison to a previous proportion of 0.36 (95% CI 0.24–0.49), the adjusted estimated proportion after adding seven additional studies was 0.19 (95% CI 0.09–0.30), which indicated a publication bias. Publication bias also existed in the estimates of worsening proportions during pregnancy (p = 0.001, N = 15), while the p-value of Egger’s test for the pooled proportion of worsening after pregnancy was 0.44 (N = 15). Sensitivity analysis did not reveal any significant changes.

## Discussion

MG is a chronic autoimmune disease of bimodal onset age with a peak occurring in young women at reproductive period. Previous studies mainly focused on the impact of muscle weakness in delivery term or the incidence of neonatal MG [13, 16, 26, 28–31], whereas only a few retrospective cohort studies have attempted to assess the factors associated with MG worsening or improvement during or after pregnancy.

To the best of our knowledge, this is the first meta-analysis to explore the risk factors for predicting the MG worsening for patients in pregnancy. Our meta-analysis revealed that the estimated proportion for MG worsening and improvement in patients during or after pregnancy is 0.36 (95% CI 0.25–0.49) and 0.28 (95% CI 0.17–0.40) respectively. From the nine studies entered the final analysis for preferential trimester, a slightly elevated worsening proportion during the third trimester was observed (0.09, 95% CI 0.02–0.19).

During and after pregnancy, distinct hormones with different concentrations may influence the immune network for MG pathogenesis in bidirectional modulation effect. In comparison to healthy controls, MG patients with anti- AChR/MuSK antibodies had low levels of galactosylated IgG2 [32]. In contrast, galactosylation and sialylation of immunoglobulin G1 and G2 increase during pregnancy and reach maximum levels in the third trimester and decrease directly in postpartum period [33]. This augmentation in IgG glycan changes has been proven to be regulated by estradiol via the overexpression of *RUNX3* [34]. Regulatory B cells (Bregs) suppress pro-inflammatory responses via interleukin 10 (IL-10), as Bregs are largely expanded by the stimulation of estrogens via the PD-1/PD-L1 pathway from early pregnancy [35, 36]. However, the pathogenic autoreactive B cells were reported to be boosted by Estrogens in the mice model of experimental autoimmune myasthenia gravis (EOMG) [37]. Progesterone, one of the most abundant hormones during pregnancy, may inhibit the formation of interleukin-17 (IL-17) producing Th17 cells and increase the frequency of regulatory T cells (Tregs) [38, 39]. Other potential mechanism included an inhibitory effect of alpha-fetoprotein on the binding of autoantibody to the AChR in MG patients [40].

As one of the mainstays in the treatments of myasthenia gravis, thymectomy has been accepted as a beneficial and effective treatment that is recommended for all patients concurrence with thymoma or refractory generalized MG with positive AChR antibodies [41–43]. For MG patients who are preparing or undergoing pregnancy, one of the most critical clinical decisions is the timing, safety and benefits of thymectomy. It has been reported that the incidence of neonatal MG was much lower if the mother had thymectomy before delivery (p = 0.03) [16]. Our meta-analysis showed concordant results that thymectomy before/during pregnancy appeared to be strong determinant of improved outcomes (OR 4.85, 95% CI 1.88–12.50, p = 0.001) and show a decreased tendency for the worsening (OR 0.55, 95% CI 0.27–1.10, p = 0.09), which can provide possible evidences for the recommendation of thymectomy for maternal MG. From an immunological point of view, thymectomy works in eliminating diversified and expanded B cells in the germinal centers thus reducing the load for fetal AChR in thymus [44]. The efficacy of thymectomy on pursuing a better prognosis of pregnancy-related myasthenia gravis can be further explored in the future, especially for the clinical outcomes in the fertile MG patients with or without thymectomy.

There are several limitations that exist in our review. As noted already, the publication bias existed in our analysis, as there was a lack of high-qualified investigations in this field such as randomized trials and longitudinal prospective cohort studies. As the objectives and methods were heterogeneous in enrolled studies, the meta-analysis was performed across different designs, various definitions for MG worsening. There was a limitation of results as we could only provide overestimation of results as many concerned factors could not be analyzed. Future prospective cohort studies are anticipated to explore the optimal managements in controlling myasthenia gravis during or after pregnancies.

## Conclusion

The MG worsening in relation to pregnancy was still unpredictable, which could occur in every period during/after pregnancy. Thymectomy before delivery did not significantly decreased the risk of worsening but was found to improve the prognosis. For the better management of MG around pregnancy, thymectomy can be recommended to pursue a better short-term prognosis.

## Supplementary Information

**Additional file 1.** Characteristics of individuals from included studies except case reports.

**Additional file 2.** Newcastle–Ottawa scale for assessing the quality of the included studies in meta-analysis.

**Additional file 3.** Myasthenia gravis status scale and definitions.

**Additional file 4.** Details of risk factors for worsening not included in the meta-analysis (single data or without original data).

**Additional file 5.** Details of clinical factors for improvement mot included in the meta-analysis (single data or without original data).

**Additional file 6.** Funnel plots of the meta-analysis before and after applying the trim-and-fill method.

## Data Availability

Datasets supporting conclusions of this article are included within the article.

## Abbreviations

MG: Myasthenia gravis
AChR: Acetylcholine receptor
MuSK: Muscle-specific kinase
OR: Odds ratio
CI: Credibility interval
MOOSE: Meta-analysis of Observational Studies in Epidemiology
EOMG: Experimental autoimmune myasthenia gravis
RNS: Repetitive nerve stimulation
Ab: Antibody
CR: Complete remission
