# Supplementary material for: Risk factors for pregnancy-related clinical outcome in myasthenia gravis: a systemic review and meta-analysis
Source: Orphanet J Rare Dis. 2022 Feb 16;17:52. doi: 10.1186/s13023-022-02205-z (PMC8848664; doi:10.1186/s13023-022-02205-z)
Supplement: Supplementary file 6 — Additional file 6. Funnel plots of the meta-analysis before and after applying the trim-and-fill method. [file 13023_2022_2205_MOESM6_ESM.pdf]

Risk factors for pregnancy-related clinical outcome in myasthenia gravis: a systemic review and meta-analysis[1]

Manqiqige Su, Xiaoqing Liu, Liang Wang, Jie Song, Zhirui Zhou, Sushan Luo†, Chongbo Zhao†

† Corresponding Authors: Sushan Luo, luosushan@fudan.edu.cn and Chongbo Zhao, MD, PhD, zhao_chongbo@fudan.edu.cn. Department of Neurology, Huashan hospital, Fudan University, No.12 Middle Wulumuqi Road, Shanghai, 200040, China.

Supplementary Information

Supplementary table 1. Characteristics of individuals from included studies except case reports

Supplementary table 2 Newcastle-Ottawa scale for assessing the quality of the included studies in meta-analysis

Supplementary table 3 Myasthenia gravis status scale and definitions

Supplementary table 4 Details of risk factors for worsening not included in the meta-analysis (single data or without original data)

Supplementary table 5 Details of clinical factors for improvement mot included in the meta-analysis (single data or without original data)

Supplementary figure. Funnel plots of the meta-analysis before and after applying the trim-and-fill method

[1] Abbreviations: MG, myasthenia gravis; AChR, acetylcholine receptor; MuSK, muscle-specific kinase; OR, odds ratio; CI, credibility interval; MOOSE, Meta-analysis of Observational Studies in Epidemiology; EOMG, experimental autoimmune myasthenia gravis; RNS, repetitive nerve stimulation; Ab, antibody; CR, complete remission

Supplementary figure. Funnel plots of the meta-analysis before and after applying the trim-and-fill method

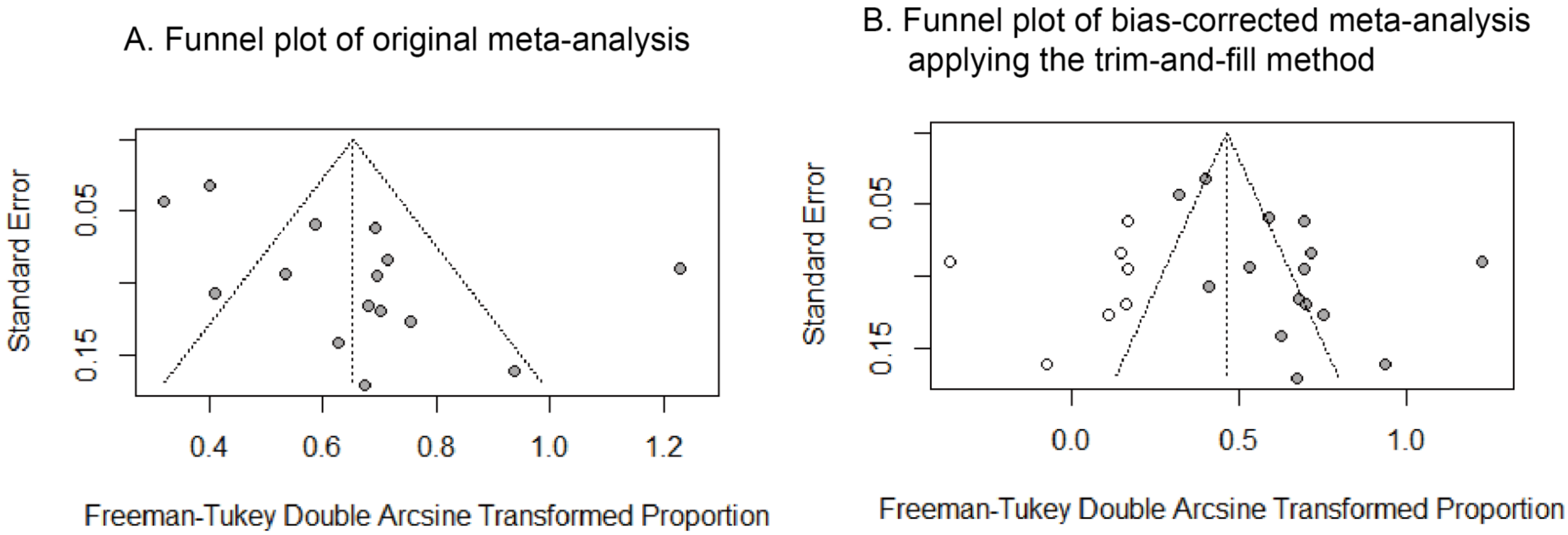

In funnel plots for the total proportions of worsening after the trim-and-fill method, seven additional studies with smaller were added to detect symmetry. In comparison to a previous proportion of 0.36 (95% CI 0.24-0.49), the adjusted estimated proportion after adding seven additional studies was 0.19 (95% CI 0.09 to 0.30). Therefore, lager worsening proportions were likely favored in the publication process, and studies with smaller proportions might be suppressed.
